# Improvement effects of different shrub sand fixation plantations on vegetation and soil in the Kubuqi Desert

**DOI:** 10.3389/fpls.2025.1688154

**Published:** 2025-11-21

**Authors:** Xinyu Guo, Guang Yang, ChenGuang Wang, Yue Ren, Xueying Han, Ning Wang, Guangpu Jia, Shi Qiao

**Affiliations:** 1 College of Desert Control Science and Engineering, Inner Mongolia Agricultural University, Hohhot, China; 2 College of Advanced Agricultural Sciences, Yulin University, Yulin, China

**Keywords:** sand-fixing plantations, vegetation diversity, soil properties, grey relational analysis, random forest model, desertification

## Abstract

Shrub plantations are a key strategy for combating desertification, yet the relative effectiveness of different species remains poorly understood. This study comprehensively evaluated the restoration effects of three dominant shrub species—*Caragana korshinskii* (CK), *Salix psammophila* (SP) and *Hedysarum scoparium* (HS)—on understory vegetation and soil properties in the Kubuqi Desert, using bare sand (BS) as the control. Shrub plantations significantly improved herbaceous vegetation diversity and soil physicochemical properties compared to BS. Specifically, CK plantations demonstrated the most pronounced positive effects, supporting the highest understory vegetation cover, species richness and soil nutrient accumulation. SP plantations were most effective in improving the physical structure of soil, resulting in the highest total porosity. Grey relational analysis weighted by principal component analysis was employed to integrate multiple indicators and provide a comprehensive evaluation, which ranked the overall restoration effectiveness as CK (0.8574) > SP (0.7790) > HS (0.6883) > BS (0.5637). Additionally, the random forest model identified biodiversity indices (particularly the Margalef index) and understory vegetation cover as the most significant drivers influencing overall restoration effectiveness, while soil available phosphorus was the only soil factor with a significant impact. These findings underscore that species selection is critical for restoration outcomes. C. *korshinskii* is recommended as a priority species for enhancing ecosystem functions in this region.

## 1 Introduction

Desertification, a form of land degradation driven by climate variability and anthropogenic factors (Burrell et al., 2020; Ngabire et al., 2023), has evolved into a global challenge demanding coordinated international efforts (United Nations Convention to Combat Desertification, U, 2017). Desertification directly affects the production and lives of 250 million people in more than 110 countries worldwide. Threatened arid lands indirectly affect about 2 billion people and cover 40% of the global land area, in both developed and developing countries (Çalişkan and Boydak, 2017). To mitigate these impacts, afforestation initiatives in arid regions have gained global traction (Yıldız et al., 2022; Guo et al., 2024; Wang et al., 2024). Scientifically designed plantation forests demonstrate significant potential in combating desertification, land degradation and climate change through systematic management. Global plantation areas are expanding at an annual rate of 3.2 million hectares and constituted 7% of global forest cover in 2015; this proportion continues to increase (United Nations Environment Programme, U, 2023).

China has experienced persistent land degradation for over six decades (Hua and Squires, 2015), with desertification currently affecting approximately 47.9 million farmers and pastoralists in the country (Wang et al., 2023). To address this challenge to ecological security and sustainable development, the Chinese government has prioritised desertification control through large-scale ecological projects (Zhang and Huisingh, 2018). For example, the Kubuqi Desert lies along the banks of the Yellow River in a region typical of northern China where agriculture and animal husbandry intersect. Its ecological location is both critically important and highly vulnerable. The management of the Kubuqi Desert directly impacts the ecological security of the middle and lower reaches of the Yellow River and the environmental quality of North China. Due to intensive human activity, extreme aridity and a low precipitation-evaporation ratio, this region has become an ecologically fragile zone with severe desertification. Historically, it was known as the ‘Sea of Death’ (Ma, 2022; UNESCO, I.C.f.C.a.S.D, 2023). Since the late 1950s, the Chinese government has designated this area as a key construction zone for the ‘Three-North Shelterbelt Forest Program’ and the ‘Beijing-Tianjin Sandstorm Source Control Project’. Early afforestation efforts primarily employed adaptable pioneer species like *Artemisia ordosica* and *Populus simonii* to rapidly stabilise shifting sands and halt desert expansion. However, many of the initially planted species exhibited low survival rates due to their inability to adapt to the extremely arid site conditions. More critically, specific deep-rooted, water-intensive tree species significantly depleted scarce groundwater resources, triggering the ecological risk arising from localised groundwater level decline (Huang et al., 2023).

In the 21st century, the Kubuqi Desert ecological restoration strategy shifted towards a more scientific and sustainable model. Building on historical lessons, current restoration efforts emphasise site-appropriate tree selection, focusing on native shrub species including *Caragana korshinskii*, *Salix psammophila* and *Hedysarum scoparium*. These shrubs exhibit superior drought tolerance, thrive in poor soils and consume relatively less water (Cheng et al., 2025; Ma et al., 2025). These sand-fixing plantations have not only improved ecological conditions but also fostered economic development in this Yellow River–adjacent region of Inner Mongolia. The desert’s successful rehabilitation earned it the designation of a ‘Global Desert Ecological and Economic Demonstration Area’ by the United Nations Environment Programme (UNEP) in 2014 (XinHua-News-Agency, 2014; Chen et al., 2022).

During the growth and development of plantation forests, understory vegetation has a significant influence on soil structure and nutrient cycling (Normand et al., 2017; Gatica-Saavedra et al., 2023). Studies have shown that the composition and diversity of understory vegetation directly or indirectly lead to changes in soil physicochemical properties (Ali et al., 2019; Han et al., 2024b). Plants also change the structure and function of soil through root distribution, canopy growth and the decomposition of apoplastic materials (Lozanova et al., 2019). Soil, as the basis for plant growth and survival, provides a location and medium for vegetation growth, and plays an inescapable role in the structure and function of plant communities (Kik et al., 2021; Sharma and Kumar, 2023). This mechanism of mutual influence and interaction between vegetation and soil is an essential link in controlling ecological processes (van der Putten et al., 2016). In arid and semi-arid regions, the development and restoration of understory vegetation soil often indicate the effectiveness of ecological reconstruction (Ayangbenro and Babalola, 2021).

Although many vegetation- and soil-related studies have been conducted on plantation forests in arid areas, most of these studies have focused on one aspect of vegetation or soil. These have included studies on the impacts of sand-fixing measures on the physical, chemical and biological properties of soil (Pérez-Corona et al., 2021; Li et al., 2023a; Liang et al., 2023), or on the composition, diversity and physiological characteristics of vegetation (Khalilimoghadam and Bodaghabadi, 2020; He et al., 2021; Qianwen et al., 2022). However, the combined benefits of vegetation and soil for plantations in sandy areas are critical. Although *C. korshinskii*, *S. psammophila* and *H. scoparium* have been widely used in ecological restoration in the Kubuqi Desert, a systematic quantitative comparison of their overall benefits to ecosystem recovery is lacking, particularly regarding their relative effectiveness in promoting understory vegetation development and improving soil.

This study investigated the understory vegetation composition, diversity, and soil physicochemical properties of three typical sand-fixing shrub plantations (*C. korshinskii*, *S. psammophila* and *H. scoparium*) in the Kubuqi Desert along the Yellow River, using adjacent bare sandy land as a control. The restoration effects were quantitatively evaluated using Grey Relational Analysis (GRA), and key influencing indicators were identified via random forest modelling. Based on the distinct functional traits of the three shrubs, we hypothesised that: 1) Their effects on understory vegetation and soil would differ significantly. Specifically, the legume *C. korshinskii*, with its dense canopy, was predicted to most effectively promote understory vegetation recovery and soil nutrient accumulation, while *S. psammophila*, with its extensive horizontal root system, would better improve shallow soil physical structure. 2) Key biotic and abiotic factors would jointly drive ecosystem restoration, with understory vegetation characteristics being more critical than soil properties. The results are expected to inform ecological restoration and plantation management in this arid, fragile ecosystem.

## 2 Materials and methods

### 2.1 Description of the study area

**Figure 1:**
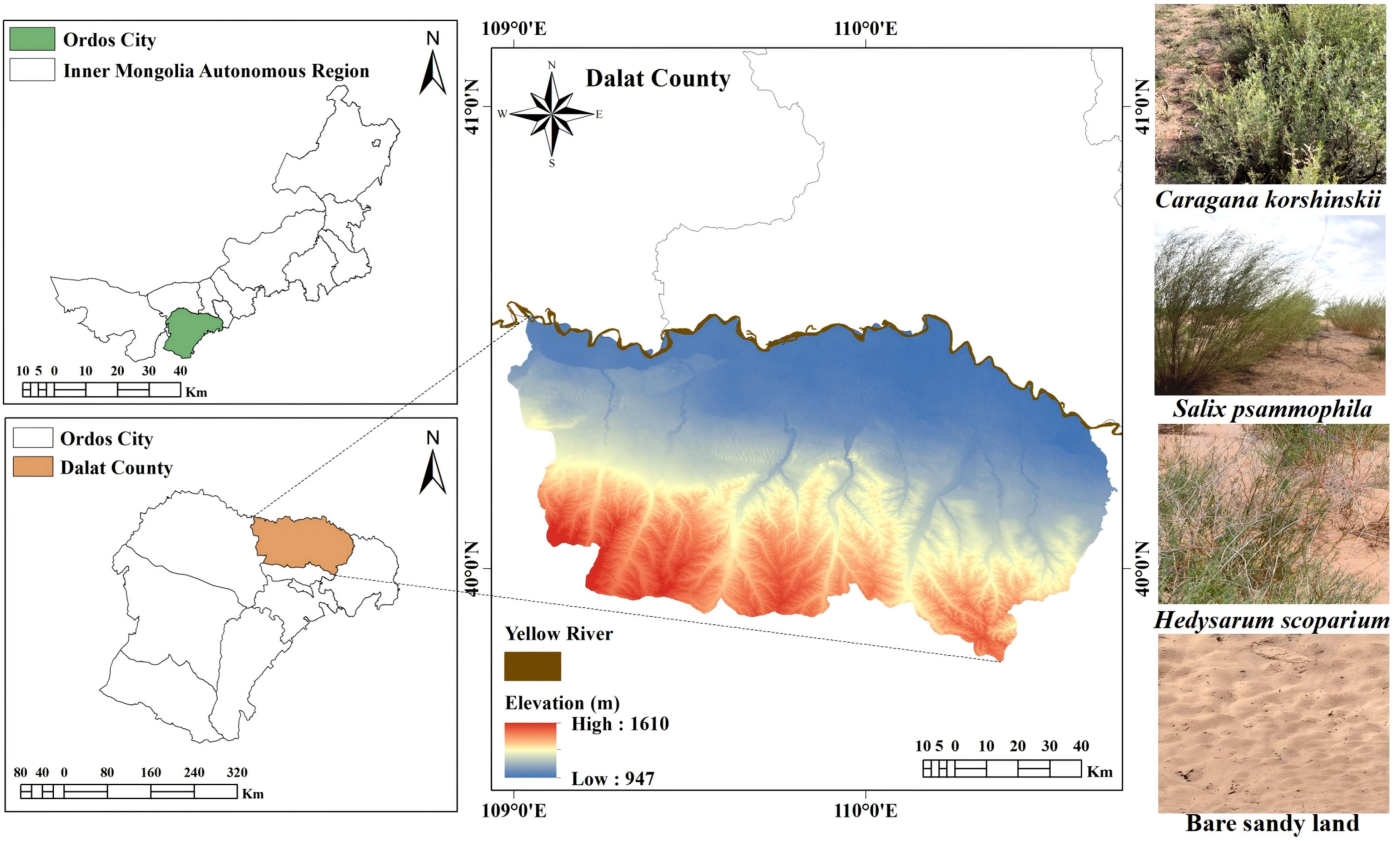
Maps of the study area. Geospatial datasets were obtained from the Geospatial Data Cloud platform (http://www.gscloud.cn), maintained by the Computer Network Information Centre of the Chinese Academy of Sciences.

The Kubuqi Desert is the seventh largest desert in China (107°00’–111°30’ E, 39°15’–40°45’ N), located at the southern edge of the middle reaches of the Yellow River. The sampling site for this study was the Kubuqi Desert Closed Reserve in Dalat County (**Figure 1**), Ordos City (109°00’–110°45’ E, 40°00’–40°30 N). The management unit of this area is the Ordos Forestation General Farm. Established in 1978, it mainly operates in the east-central part of the Kubuqi Desert, which is one of the key construction units of the ‘Three Norths’ protection forests established by the Chinese government (Ordos-Daily-News, 2024). The area has a temperate continental climate, which is characterised by high winds, sand, dryness, and little rain. The summers are hot and short, and the winters are cold and long, with drastic temperature changes. The average annual precipitation is 240–360 mm, with most occurring in July–September, accounting for about 61% of the annual total. The average number of sunshine hours each year is 3,159.4 hours, and the average annual frost-free period is 130–140 days (Li et al., 2023b; Yongsheng et al., 2023; Baidu-Library, 2024; Han et al., 2024a). The main soil type in the sampling area is gray desert soil. The vegetation is primarily composed of sand-dwelling shrubs, subshrubs, and herbaceous plants, such as *Caragana korshinskii*, *Artemisia ordosica*, and *Agriophyllum squarrosum*. In recent years, the Chinese government has increasingly focused on desertification control. Following the launch of key ecological programs like the ‘Three-North Shelterbelt Project’ and the Yellow River Basin conservation/development strategy, extensive sand-fixing plantations have been installed in the study area, resulting in substantial ecological restoration and environmental improvement (XinHua-News-Agency, 2014; Li et al., 2023a; UNESCO, I.C.f.C.a.S.D, 2023).

### 2.2 Experimental design and site description

The field survey and sample collection were carried out in August 2024 at the Ordos Forestation General Farm in Dalat County. A spatially nested sampling design was employed to evaluate the three dominant sand-fixing shrub plantations (*C. korshinskii*, CK; *S. psammophila*, SP; *H. scoparium*, HS) against a bare sand (BS) control. Critically, prior to plantation establishment, all selected plots were shifting bare sand areas with no history of alternative land use, ensuring highly consistent initial site conditions, including soil texture. For each of these four land types, three independent replicate sites were established (e.g., named CK-1, CK-2 and CK-3 for C. *korshinskii*), resulting in a total of 12 independent study sites (**Table 1**). The plantation areas had consistent site conditions. The distances between different types of plots in the plantation area were less than 1 km, with relatively flat terrain (slope < 2°), eliminating potential confounding effects of slope and aspect on vegetation and soil characteristics. The density (1600–1733 trees·hm^–2^) and the age (26–29 a) of the plantations were similar, and the understory plant communities had naturally regenerated.

**Table 1:** Basic information for the sample plots.

Plot type	Plot symbol	Forest age (a)	Silvicultural density (trees·hm^–2^)	Understory vegetation coverage (%)
*Caragana korshinskii*	CK-1	28	1,600	34.9 ± 2.18
	CK-2	28	1,600	
	CK-3	28	1,600	
*Salix psammophila*	SP-1	28	1,641	26.13 ± 1.71
	SP-2	29	1,644	
	SP-3	29	1,644	
*Hedysarum scoparium*	HS-1	26	1,689	20.2 ± 1.26
	HS-2	27	1,733	
	HS-3	27	1,733	
None (Bare sand)	BS-1	–	–	11.27 ± 1.2
	BS-2	–	–	
	BS-3	–	–	

Data are mean ± standard deviation.

### 2.3 Vegetation and soil sampling

Within each of the 12 independent sites, a 30 m × 30 m plot was established for intensive sampling. The understory vegetation community was investigated using the quadrat method (1 × 1 m) and the five-point sampling method. Within each quadrat, vegetation coverage was quantitatively estimated using a 10 × 10 grid (100 squares), by counting the number of squares in which vegetation was vertically projected. The plant species, height, cover and frequency within the quadrat were recorded. For soil analysis, three soil sampling points were evenly located along the diagonal of each plot. At each point, soil was sampled using the profile method to a depth of 60 cm, with samples collected at 20 cm intervals (0–20, 20–40, 40–60 cm). This design resulted in a total of 108 soil samples (4 land types × 3 replicate sites × 3 sampling points × 3 depths = 108). From these, undisturbed soil cores were collected at each depth interval using a 100 cm³ core sampler to determine soil water content, bulk density and porosity. Additional soil samples were collected at each point and depth, placed in sealed bags, and transported to the laboratory.

### 2.4 Laboratory analysis

In the laboratory, soil samples were air-dried in the dark and sieved to remove plant residue and rocks. The soil particle size distribution was determined using a Fritsch Analysette 22 MicroTecPlus laser particle size analyser (Fritsch GmbH, Germany). Nutrient analyses were performed as follows. Total nitrogen, Kjeldahl digestion method using a H_2_SO_4_-HClO_4_ mixture; total phosphorus, H_2_SO_4_-HClO_4_ digestion followed by molybdenum-antimony colourimetry; total potassium, acid digestion with flame photometric detection; available nitrogen, alkali diffusion method; available phosphorus, (0.5 mol/L NaHCO_3_-H_2_SO_4_ extraction followed by molybdenum-antimony colourimetry; available potassium (1 mol/L CH_3_COONH_4_ extraction and flame photometric analysis. These soil nutrient determination methods were from the Soil Environmental Protection Standard issued by the People’s Republic of China (National-Forestry-and-Grassland-Administration, 1999).

### 2.5 Vegetation characteristic indicators

The importance value is used as an indicator of population dominance (Mensah et al., 2020). This was used to understand the distribution pattern and functional status of herbaceous vegetation species under different plantations. Based on previous species diversity research results (Kim et al., 2017; Mensah et al., 2020; Haq et al., 2024), the Simpson, Shannon-Wiener, Pielou and Margalef indexes were chosen to analyse the characteristics of species diversity of the vegetation communities in different types of sand-fixing plantations.

### 2.6 Comprehensive evaluation

#### 2.6.1 Normalisation of indicators

To facilitate the comparison and weighting of indicators of different units or magnitudes, the affiliation function (Equation 1) was used to normalise the vegetation and soil indicators (Hemati et al., 2020; Guo et al., 2025).

(1) $$F(\text{x})=E/(1+{\left(X_{i}/X_{0}\right)}^{i})$$

Here, *E* is the maximum membership degree of the evaluation index with the value 1; *X_i_* is the value of each evaluation index; *X_0_* is the average value of each evaluation index; *i* is the slope of the equation, where the value –2.5 indicates that the index has a positive effect, and 2.5 means that the index has a negative effect.

#### 2.6.2 Principal component analysis assignment of indicator weights

Principal component analysis (PCA) was performed using SPSS 26.0 to quantify the relative information contribution of each index. Principal components with eigenvalues ≥ 1 and a cumulative contribution rate ≥ 85% were retained. The data input for the PCA was a matrix of site-level means. Specifically, all vegetation and soil measurements from the sub-samples within each of the 12 independent sites were averaged to produce a single, composite profile for that site. This approach ensured the statistical independence of the data points used in the ordination. The variance contribution rate of each variable from the PCA was used to calculate its weight for the subsequent GRA, transforming the evaluation from equally weighted to weighted-comprehensive. Indicator weights were assigned based on their contributions to the variance.

#### 2.6.3 Grey relational analysis

Grey relational analysis (GRA) was used to quantitatively evaluate the restoration effects of different types of plantations. GRA is a method that determines the strength of the relationship between evaluation objects and a reference object by calculating the similarity (degree of association) of the geometric shapes between data sequences. It is suitable for comprehensive evaluation and factor analysis problems with small samples, multiple indicators and limited information (Lu et al., 2023). This concept was originally proposed by Deng Julong, a professor in the discipline of control science and engineering (Julong, 1989). It primarily constructs standardised data sequences and reference sequences, calculates the grey relational coefficients and degrees of the evaluated objects relative to the reference object, and ranks the evaluated objects or analyses the importance of factors based on the degrees of correlation, thereby providing a basis for decision-making. The closer the evaluation sequence is to the reference sequence, the higher its comprehensive ranking and the better the evaluation object is (Chen, 2023). This process is described by Equations 2-6.

1. Establishment of evaluation object sequence and reference sequence

The reference object (Equation 2)

(2) $$X_{t}=\{X_{t}(1),X_{t}(2),\ldots ,X_{t}(\text{n})\}$$

The sequence of evaluation objects (Equation 3)

(3) $$X_{\text{p}}=\{X_{p}(1),X_{p}(2),\ldots ,X_{p}(\text{n})\}$$

Here, p =1, 2,…, m.

2. The grey relation factor (Equation 4)

(4) $$\xi_{p}(h)=\frac{\min_{p}\min_{\text{h}}|X_{t}(h)-X_{p}(h)|+\rho \max_{p}\max_{h}|X_{t}(h)-X_{p}(h)|}{\left||X_{t}(h)-X_{p}(h)\;|+\rho \max_{p}\max_{h}|X_{t}(h)-X_{p}(h)|\right|}$$

Here, *│X_t_(h)-X_p_(h)│* represents the absolute difference between data sequences X_t_ and X_p_ at a particular measurement point h; the term *min_p_ min_h_│X_t_(h)-X_p_(h)│* represents the minimum absolute difference corresponding to factor p = 1,2,…,m at the same point h =1,2,…, which is called the second-order minimum difference; *max_p_ max_h_│X_0_(h)-X_p_(h)│* represents the second-order maximum difference; and ρ is a resolution coefficient with a value between 0 and 1 that is usually set to 0.5.

(3) Grey relevance (Equations 5, 6)

(5) $$\gamma_{\text{p}}=\frac{1}{n}\sum_{\text{h}=1}^{n}\xi_{p}(\text{h})$$

(6) $$R_{\text{p}}=\sum_{\text{h}=1}^{n}\xi_{p}(\text{h})\cdot K_{\text{p}}$$

Here, *γ_p_* is the equal weight relevance; n is the number of evaluation indicators determined; *R_p_* is the weighted relevance; and *K_p_* is the weight of the index.

### 2.7 Random forest modelling

The random forest model is a powerful ensemble machine learning algorithm that constructs a multitude of decision trees during training and outputs the mean prediction of the individual trees for regression tasks (Rigatti, 2017; Charbuty and Abdulazeez, 2021). In this study, a random forest regression was employed to predict the comprehensive restoration score (from GRA) and identify the most influential drivers among the measured vegetation and soil properties. All analyses were done in the R 4.4.2 environment, primarily using the randomForest package for model construction, the caret package for parameter optimisation and the dplyr package for data processing. The data were divided in a 7:3 ratio, with 70% of the samples used as the training set for model construction and 30% of the samples used as the independent test set to verify the model performance. The model parameter optimisation was achieved through five-fold cross-validation. The key parameter mtry (the number of randomly selected variables in each tree) was optimised within the range of 3 to 15 in random forests, and the optimal parameter combinations were finally determined. To assess the statistical significance of variable importance, a permutation test was applied wherein the target variable (GRA) was randomly permuted 1000 times. For each permutation, the model was retrained, and the permutation importance value was calculated. The p-value for each variable was then determined based on the proportion of permutation iterations where the permuted importance exceeded the original importance. Significance levels were assigned as follows: *** for *p* < 0.001, ** for *p* < 0.01 and * for *p* < 0.05.

### 2.8 Data processing and analysis

Statistical analyses were performed using SPSS 26.0, with figures and the study area map generated using OriginPro 2021 and ArcGIS 10.6, respectively. Prior to modelling, the normality of continuous variables was assessed using the Shapiro-Wilk test, and homogeneity of variances was confirmed with Levene’s test. For variables violating normality (P ≤ 0.05), data transformations (natural log, square root, or reciprocal) were applied. A generalised linear mixed model was employed to assess the effects of shrub type and soil depth (fixed effects) on vegetation and soil properties, with ‘Plot symbol’ (e.g., CK-1, CK-2, CK-3) as a random effect to account for repeated measurements. Appropriate link functions and error distributions were selected for continuous response variables. For significant fixed effects (α = 0.05), *post hoc* pairwise comparisons were conducted using the least significant difference (LSD) method. All tests used Type III sums of squares, and results are presented as mean ± standard deviation.

## 3 Results

### 3.1 Understory vegetation characteristics

#### 3.1.1 Vegetation species composition and importance values

A total of 20 plant species belonging to seven families and 17 genera were found in the 60 vegetation quadrats within the 12 sample plots (**Table 2**). There were six species of Asteraceae, accounting for 30% of the total species, of which *Artemisia* accounted for 20% of the total species; four species each of Fabaceae and Amaranthaceae, each accounting for 20% of the total species; and three species of Poaceae, accounting for 15% of the total species. There were 14 genera and 17 species in these four families, accounting for 85% of the total species. The other plants were from individual families, genera and species, indicating that these four families played an important role in the sandy plantation ecosystem along the Yellow River and had good adaptability to the natural environment in the study area.

**Table 2:** Plant community composition and important values.

Family	Genus	Species	Sample plot			
			CK	SP	HS	BS
Asteraceae	*Artemisia*	*Artemisia frigida*	2.19	–	–	–
Asteraceae	*Artemisia*	*Artemisia desertorum*	12.98	17.54	28.5	20.26
Asteraceae	*Artemisia*	*Artemisia capillaris*	1.95	2.03	4.11	–
Asteraceae	*Artemisia*	*Artemisia annua*	3.1	–	–	–
Asteraceae	*Aster*	*Aster hispidus*	–	–	17.66	15.25
Asteraceae	*Ixeris*	*Ixeris polephala*	–	–	6.43	1.56
Fabaceae	*Lespedeza*	*Lespedeza bicolor*	17.6	8.12	3.88	6.82
Fabaceae	*Sophora*	*Sophora alopecuroides*	3.71	–	–	–
Fabaceae	*Astragalus*	*Astragalus laxmannii*	3.52	2.7	3.61	–
Fabaceae	*Oxytropis*	*Oxytropis racemosa*	–	1.58	–	–
Amaranthaceae	*Agriophyllum*	*Agriophyllum squarrosum*	5.25	3.66	2	15.87
Amaranthaceae	*Bassia*	*Bassia scoparia*	3.39	–	–	–
Amaranthaceae	*Salsola*	*Salsola collina*	5.18	26.9	–	–
Amaranthaceae	*Suaeda*	*Suaeda glauca*	–	2.11	–	–
Poaceae	*Setaria*	*Setaria viridis*	12.17	8.25	16.8	35.52
Poaceae	*Stipa*	*Stipa caucasica subsp. glareosa*	5.36	7.74	4.53	–
Poaceae	*Agropyron*	*Agropyron mongolicum*	1.83	4.17	5.41	4.72
Zygophyllaceae	*Tribulus*	*Tribulus terrestris*	21.77	–	7.07	–
Euphorbiaceae	*Euphorbia*	*Euphorbia humifusa*	–	12.35	–	–
Bignoniaceae	*Incarvillea*	*Incarvillea sinensis*	–	2.85	–	–
Number of species			14	13	11	7
Number of annuals			5	5	4	4
Number of perennials			9	8	7	3
Average understory vegetation cover (%)			34.9	26.13	20.2	11.27

CK, *Caragana korshinskii* plantation; SP, *Salix psammophila* plantation; HS, *Hedysarum scoparium* plantation; BS, bare sand.

The total number of plant species and perennials in the different sites was in the order CK > SP > HS > BS. There were four dominant species in the CK plantations: *Tribulus terrestris*, *Setaria viridis*, perennial *Lespedeza bicolor* and *Artemisia desertorum*. There were three dominant species in the SP plantations: *Salsola collina*, *Euphorbia humifusa* and perennial *A. desertorum*. The HS plantation also had three dominant species: annual *S. viridis* and *Aster hispidus*, and perennial *A. desertorum*. The average cover of vegetation under different plantations was in the order CK > SP > HS > BS. There were more dominant species in the understory vegetation of CK plantations compared to the other plantations. The species composition of the SP and HS plantations was relatively simple.

#### 3.1.2 Vegetation species diversity characteristics

The Shannon–Winner, Simpson, Margalef and Pielou indexes were in the order CK > SP > HS > BS (**Table 3**). There was no significant difference in the Shannon–Winner index between the plantation plots (*p* > 0.05), but there was a significant difference between the plantation and BS plots (*p* < 0.05). There was no significant difference in the Pielou index among all plots (*p* > 0.05). The Simpson and Margalef indexes were significantly different among all plots (*p* < 0.05).

**Table 3:** Characteristics of community diversity in different sand fixation plantations.

Community diversity indicators	Sample plot			
	CK	SP	HS	BS
Shannon–Winner index	0.87 ± 0.01 A	0.85 ± 0.01 A	0.84 ± 0.01 A	0.77 ± 0.02 B
Simpson index	2.28 ± 0.04 A	2.19 ± 0.06 B	2.05 ± 0.02 C	1.63 ± 0.04 D
Pielou index	0.87 ± 0.02 A	0.86 ± 0.02 A	0.85 ± 0.01 A	0.84 ± 0.02 A
Margalef index	2.37 ± 0.09 A	2.25 ± 0.01 B	2.07 ± 0.03 C	1.35 ± 0.03 D

Data are given as mean ± standard deviation. Different capitalised letters indicate significant differences between sample plots (*p* < 0.05).

### 3.2 Understory soil characteristics

#### 3.2.1 Soil particle size characteristics

Different types of plantations had significant effects on the particle composition of sandy soil (**Table 4**). Soil particles were graded according to the US Department of Agriculture’s Soil Texture Grading Criteria. Compared with BS, the soil clay content increased by 0.05%–0.51%, the soil silt content increased by 0.33%–0.71% and the soil sand content decreased by 0.41%–1.16% in the different plantations. The clay, silt and very fine sand content followed the order CK > SP > HS > BS, and the content of fine sand, medium sand, coarse sand and very coarse sand followed the order BS > HS > SP > CK. There were significant differences in clay, silt and very fine sand content within the same soil layer (*p* < 0.05), no significant difference between medium sand and coarse sand content between SP and HS (*p* > 0.05) and no significant difference in very coarse sand content among different plots (*p* > 0.05). Except for fine sand, there were significant differences in all soil particles among different soil layers (*p* < 0.05), and there was no significant difference in fine sand in the 20–40 cm and 40–60 cm soil layers of all sample plots (*p* > 0.05). In different soil layers, the clay, silt and very fine sand content followed the order 0–20 cm > 20–40 cm > 40–60 cm, and the content of medium sand, coarse sand and very coarse sand particles followed the order 0–20 cm < 20–40 cm < 40–60 cm.

**Table 4:** Distribution of soil particles in different sand fixation plantations.

Soil particle size	Sample plot	Soil depth (cm)		
		0–20	20–40	40–60
Clay ( < 0.02 mm)	CK	0.63 ± 0.05 Aa	0.48 ± 0.02 Ab	0.21 ± 0.03 Ac
	SP	0.42 ± 0.02 Ba	0.26 ± 0.03 Bb	0.15 ± 0.03 Bc
	HS	0.31 ± 0.03 Ca	0.18 ± 0.03 Cb	0.08 ± 0.02 Cc
	BS	0.12 ± 0.03 Da	0.07 ± 0.02 Db	0.03 ± 0.02 Dc
Silt (0.02 mm ~ 0.05mm)	CK	1.17 ± 0.03 Aa	0.95 ± 0.02 Ab	0.78 ± 0.04 Ac
	SP	0.94 ± 0.03 Ba	0.81 ± 0.02 Bb	0.63 ± 0.01 Bc
	HS	0.85 ± 0.03 Ca	0.73 ± 0.03 Cb	0.55 ± 0.02 Cc
	BS	0.52 ± 0.02 Da	0.24 ± 0.02 Db	0.19 ± 0.02 Dc
Very fine sand (0.05 mm ~ 0.1 mm)	CK	5.76 ± 0.20 Aa	4.93 ± 0.20 Ab	3.73 ± 0.34 Ac
	SP	4.84 ± 0.13 Ba	3.76 ± 0.29 Bb	2.68 ± 0.20 Bc
	HS	3.85 ± 0.16 Ca	3.01 ± 0.14 Cb	2.13 ± 0.16 Cc
	BS	2.04 ± 0.23 Da	1.51 ± 0.20 Db	0.95 ± 0.04 Dc
Fine sand (0.1 mm ~ 0.25 mm)	CK	73.17 ± 0.45 Ba	69.78 ± 0.43 Bb	70.18 ± 0.58 Ab
	SP	73.47 ± 0.51 Ba	69.87 ± 0.48 Bb	70.37 ± 0.39 Ab
	HS	74.53 ± 0.45 Aa	70.37 ± 0.34 Ab	70.40 ± 0.29 Ab
	BS	74.83 ± 0.52 Aa	70.85 ± 0.36 Ab	70.56 ± 0.44 Ab
Medium sand (0.25 mm ~ 0.5 mm)	CK	16.64 ± 0.21 Cc	19.72 ± 0.38 Cb	20.67 ± 0.36 Ca
	SP	17.01 ± 0.41 Bc	20.82 ± 0.27 Bb	21.38 ± 0.29 Ba
	HS	17.11 ± 0.44 Bc	21.16 ± 0.20 Bb	21.78 ± 0.19 Ba
	BS	18.65 ± 0.47 Ac	22.08 ± 0.37 Ab	22.52 ± 0.32 Aa
Coarse sand (0.5 mm ~ 1 mm)	CK	2.12 ± 0.15 Cc	3.54 ± 0.25 Cb	3.76 ± 0.16 Ca
	SP	2.79 ± 0.17 Bc	3.85 ± 0.17 Bb	4.11 ± 0.19 Ba
	HS	2.81 ± 0.18 Bc	3.90 ± 0.19 Bb	4.36 ± 0.16 Ba
	BS	3.26 ± 0.17 Ac	4.58 ± 0.32 Ab	5.03 ± 0.16 Aa
Very coarse sand (1 mm ~ 2 mm)	CK	0.51 ± 0.02 Ac	0.60 ± 0.02 Ab	0.67 ± 0.05 Aa
	SP	0.53 ± 0.02 Ac	0.63 ± 0.01 Ab	0.68 ± 0.02 Aa
	HS	0.54 ± 0.02 Ac	0.65 ± 0.02 Ab	0.70 ± 0.02 Aa
	BS	0.58 ± 0.02 Ac	0.67 ± 0.02 Ab	0.72 ± 0.02 Aa

Data are given as mean ± standard deviation. Different capitalised letters in the same soil depth indicate significant differences between sample plots, while different lowercase letters in the same sample plot indicate significant differences between different soil depths (P < 0.05). The same below.

#### 3.2.2 Soil water content, bulk density and porosity characteristics

**Figure 2:**
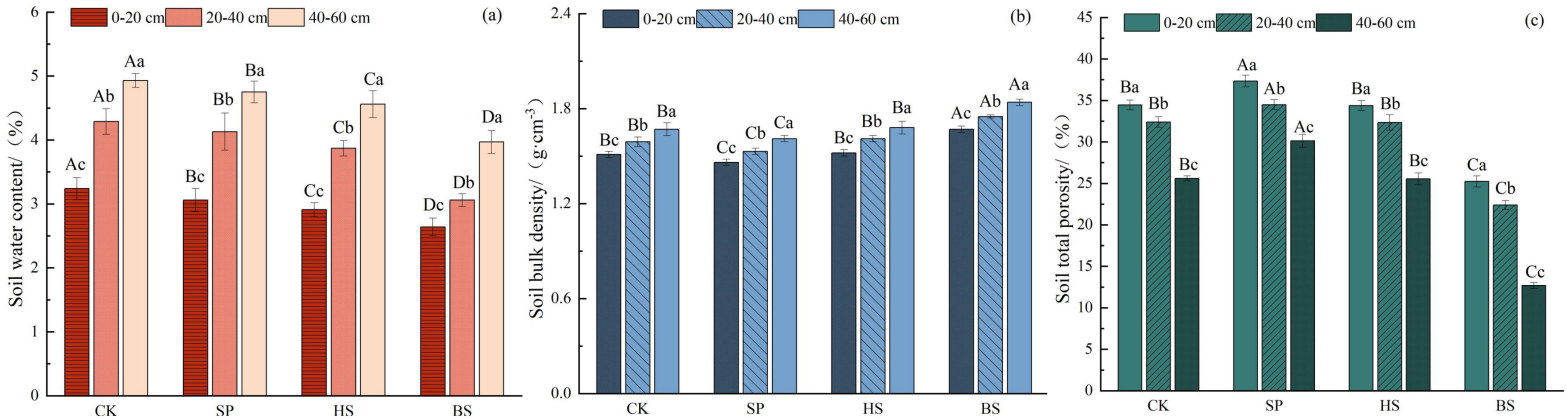
Soil physical propertiesin different sandfixation plantations. **(a)** Soil water content, **(b)** Soil bulk density, and **(c)** Soil total porosity.

The construction of plantations in sandy land significantly reduced soil bulk density, and increased soil water content and porosity (**Figure 2**). The soil water content followed the order BS < HS < SP < CK, the soil bulk density was SP < CK < HS < BS, and the soil total porosity was SP > CK > HS > BS. Soil water content and bulk density in different soil depths followed the order 0–20 cm < 20–40 cm < 40–60 cm, and soil porosity was 0–20 cm > 20–40 cm > 40–60 cm. There was no significant difference in soil bulk density and porosity between CK and HS at the same soil depth (*p* > 0.05). The above indicators showed significant differences between soil layers in the same area (*p* < 0.05).

#### 3.2.3 Soil nutrient characteristics

**Figure 3:**
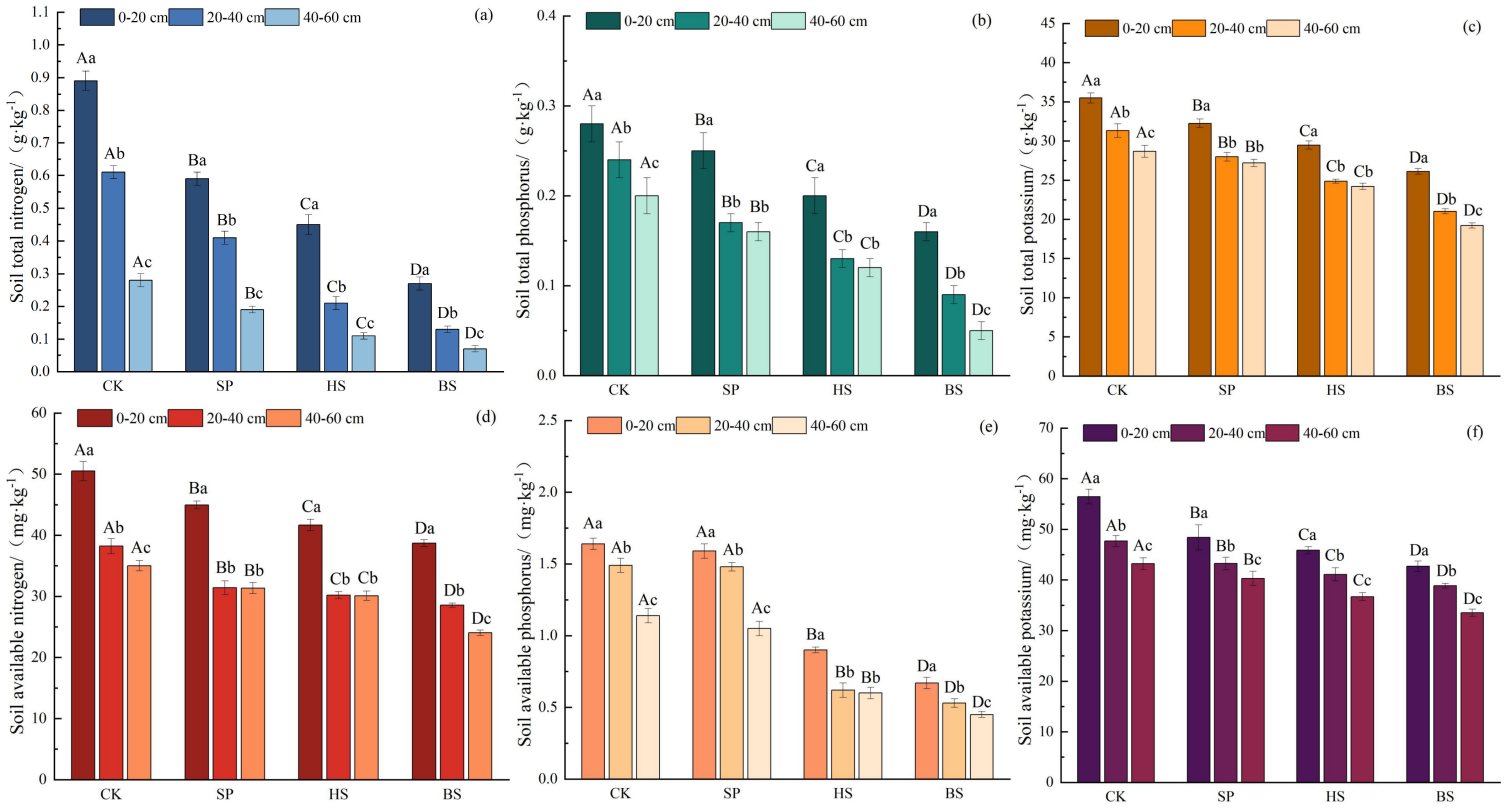
Soil nutrient characteristics in different sand fixation plantations. **(a–c)** Total nitrogen, phosphorus, and potassium in the soil; **(d–f)** Available nitrogen, phosphorus, and potassium in the soil.

In general, the soil nutrient pattern followed the order CK > SP > HS > BS (**Figure 3**). The construction of sand-fixing plantations effectively improved the nutrient content of sandy soil. In the same plot, the soil nutrient content of different soil layers followed the order 0–20 cm > 20–40 cm > 40–60 cm, with the soil nutrients in sandy soil mainly concentrated in the 0–20 cm layer. There were significant differences in soil total nitrogen and available potassium in different plots and at different soil depths (*p* < 0.05). There were significant differences in soil total phosphorus, total potassium and available nitrogen among different plots (*p* < 0.05), and there was no significant difference in above indicators between the 20–40 cm and 40–60 cm soil layers of the SP and HS plantations (*p* > 0.05). There was no significant difference in soil available phosphorus content between the CK and SP plantations (*p* < 0.05), but these contained significantly higher levels than HS and BS. In HS, there was no significant difference in soil nutrients between the 20–40 cm and 40–60 cm soil layers (*p* > 0.05), whereas the available phosphorus content in other plots was significantly different across all soil layers.

### 3.3 Grey relational analysis

In GRA, equal weights cannot objectively reflect comprehensive evaluation results because they do not account for the varying degrees of influence among indicators. Therefore, a weighted analysis considering indicator weights is typically required. PCA was performed on the normalised vegetation and soil system indicators (**Table 5**), yielding two principal components with eigenvalues >1 and a cumulative contribution rate of 88.123%. The indicator weights for GRA were determined based on the variance contribution rate of each indicator. The evaluation results of the equal weights and weighted GRA (**Table 6**) followed the order CK > SP > HS > BS.

**Table 5:** Principal component analysis of grey relational analysis.

Indicator	Principal component		Communality	Weight
	PC-1	PC-2		
Shannon–Winner index	0.908	0.269	0.896	0.0508
Simpson index	0.921	0.355	0.975	0.0553
Pielou index	0.634	–0.091	0.41	0.0233
Margalef index	0.843	0.497	0.958	0.0544
Understory vegetation coverage	0.893	0.368	0.933	0.0529
Number of species	0.895	0.423	0.98	0.0556
Dominant species importance value	–0.115	–0.751	0.578	0.0328
Number of perennial species	0.888	0.431	0.975	0.0553
Clay	0.928	–0.257	0.927	0.0526
Silt	0.982	–0.047	0.967	0.0549
Sand	–0.982	0.132	0.981	0.0557
Soil water content	0.044	0.984	0.97	0.0550
Soil bulk density	–0.875	0.213	0.812	0.0461
Soil total porosity	0.844	–0.154	0.737	0.0418
Soil total nitrogen	0.894	–0.363	0.932	0.0529
Soil total phosphorus	0.949	–0.192	0.938	0.0532
Soil total potassium	0.967	–0.162	0.961	0.0545
Soil available nitrogen	0.799	–0.516	0.904	0.0513
Soil available phosphorus	0.92	–0.001	0.846	0.0480
Soil available potassium	0.892	–0.387	0.946	0.0537
Characteristic root	14.37	3.254	–	–
Variance contribution rate (%)	71.852	16.271	–	–
Accumulated variance contribution rates (%)	71.852	88.123	–	–

**Table 6:** Grey relevance evaluation results.

Sample plot	Equal weight relevance		Weighted relevance	
	Relevance ranking	Ranking	Relevance	Ranking
CK	0.8539	1	0.8574	1
SP	0.7818	2	0.7790	2
HS	0.6982	3	0.6883	3
BS	0.5812	4	0.5637	4

### 3.4 Random forest model

**Figure 4:**
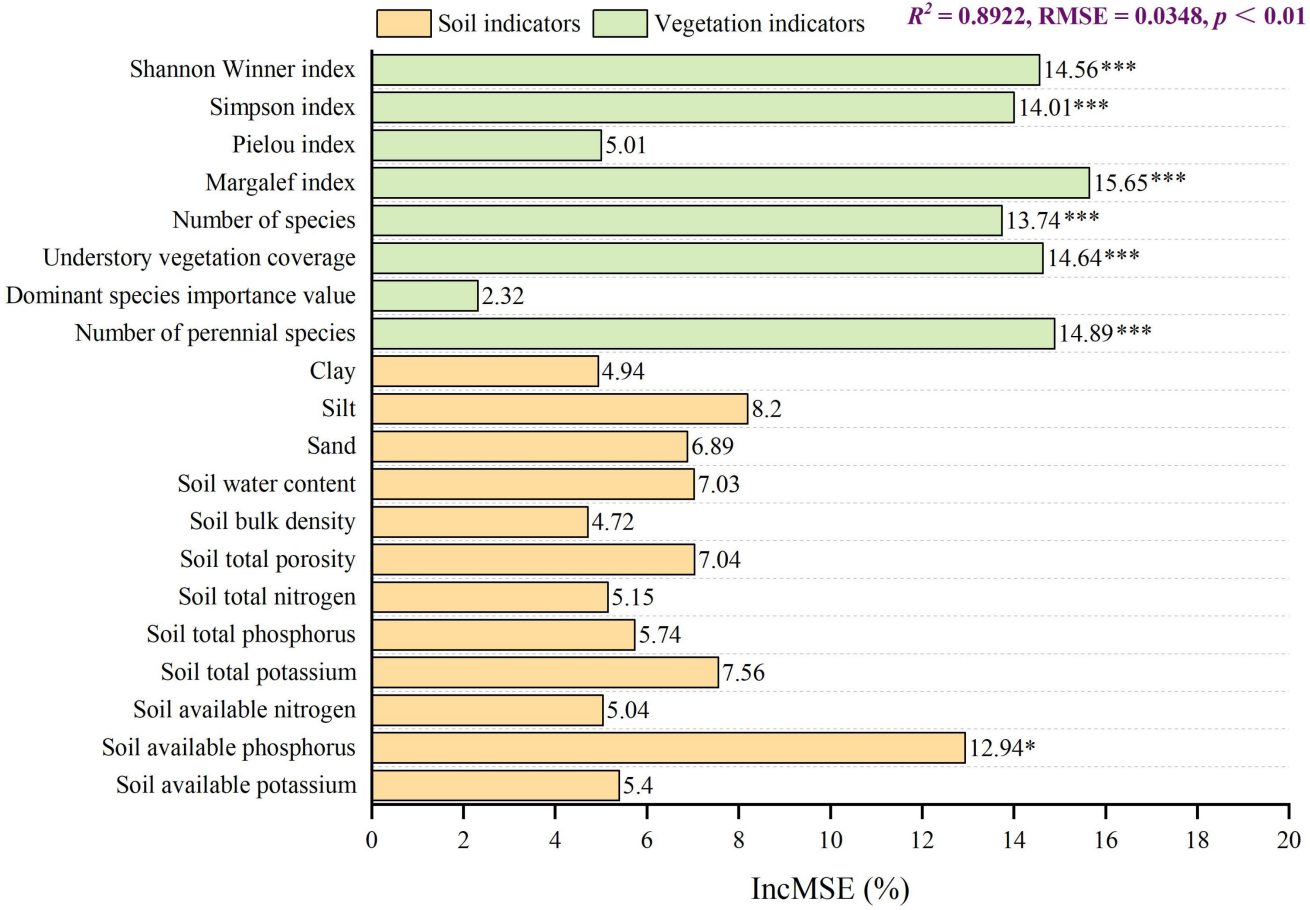
Influence of vegetation and soil indicators on GRA. ***p < 0.001, *p < 0.05.

The random forest model was employed to analyse the impact of vegetation and soil indicators on the GRA results (**Figure 4**). The model exhibited high predictive accuracy and reliability, with an R² of 0.8922 (*p* < 0.01, permutation test) and an RMSE of 0.0348. Among the explanatory variables, biodiversity indicators were identified as the most influential drivers of the GRA. Specifically, the Margalef index (reflecting species richness) demonstrated the highest variable importance (%IncMSE = 15.65), closely followed by the number of perennial species (%IncMSE = 14.89) and understory vegetation coverage (%IncMSE = 14.64). The Shannon-Wiener index, Simpson index and total number of species also exhibited substantial and statistically significant influences (*p* < 0.001). In contrast, soil factors showed limited effects; only soil available phosphorus (%IncMSE = 12.94) reached significance (*p* < 0.05), while other soil properties were non-significant. These results indicate that for ecological restoration in sandy regions, priority should be given to selecting tree species that enhance understory vegetation coverage and promote species diversity. Additionally, future forest management practices should place greater emphasis on soil available phosphorus.

## 4 Discussion

### 4.1 The effects of desert area plantations on vegetation and soil

Deserts are typically ecologically fragile areas. Vegetation characteristics and soil physicochemical properties are indicators of the development and evolution of the plantation vegetation soil system. Numerous relevant studies have shown that vegetation and soil can be effectively improved through scientific plantation construction on degraded land, particularly in shrub forests. These studies cover typical degraded areas in northwestern China, as well as degraded lands in the Mediterranean region and reclaimed desertified areas in Iran (Kooch and Noghre, 2020; Cheng and Wang, 2022; Navarro-Perea et al., 2022). The present study reaffirmed this conclusion and further quantified the positive effects of sand-fixing plantations of *C. korshinskii* (CK), *S. psammophila* (SP) and *H. scoparium* (HS) in the Kubuqi Desert on vegetation diversity and soil properties.

Ecological reconstruction in nutrient-poor and moisture-limited desert areas is a long and complex process. As plantations grow, the understory vegetation community gradually changes. The development of the root system, the decomposition of deadfall and the redistribution of natural resources such as rainfall and sunlight by the canopy change the temperature and microenvironment of the soil, improve its water- and fertiliser-retention capacity, and slow the evaporation of soil water and the decomposition of organic matter (Li et al., 2024; Lyu et al., 2025). This creates basic living conditions for a wide range of herbaceous plants that would not otherwise survive on bare sand, initiating the process of ecological succession. The soil then feeds nutrients and water back to the vegetation, forming a mutually reinforcing and inseparable relationship between vegetation and soil (van der Putten et al., 2016). This study found that *Artemisia* plants are well adapted to the study area, mainly because their physiological characteristics effectively reduce transpirational water loss. Some species can also become dormant during drought and resume growth when water conditions improve. *Artemisia* also have a well-developed root system and is generally highly tolerant of barrenness, enabling it to survive and thrive in conditions of limited nutrients and water (Pang et al., 2022; Atamian and Funk, 2023).

In this study, the analysis of the spatial distribution of soil physicochemical properties in the 0–60 cm soil layer revealed that all the plantations exhibited the highest content of fine particulate matter and nutrients in the 0–20 cm soil layer. This was in agreement with the findings of related studies by Li et al (Li et al., 2017, 2022). This may have been because the branches and leaves of the vegetation trapped fine dust particles in the air, which were then replenished on the soil surface through rainfall. Soil fine particles are the key sources of nutrient enrichment. None of the plantations selected for the present study had a significant effect on the very coarse sand content of the soil, probably because they were shrubs. Shrub forests only reduce wind speed at a height of 0–2 m (Pan et al., 2021) and have a limited inhibitory effect on the leapfrog movement of very coarse sand. Winds mainly carry medium and fine sands. The movement of very coarse sands requires wind speeds in excess of those needed for common wind erosion, making them difficult to redistribute via wind. Fine shrub roots form root-soil complexes mainly with < 0.25 mm particles (Lai et al., 2016), whereas very coarse sand is difficult to entangle by roots or bind by secretions because of its large particle size.

### 4.2 Comprehensive evaluation of different plantations

The evaluation results based on GRA indicated a clear hierarchy of restoration effectiveness: CK > SP > HS. Previous studies in high-cold desert areas and the severely soil-eroded Loess Plateau have confirmed that CK promotes ecological restoration in arid and semi-arid regions and exhibits extraordinary adaptability to drought stress (Li et al., 2016; Zhao et al., 2021). The advantages of CK may additionally stem from its ability to synergistically improve the chemical and physical microhabitats of soil. As a nitrogen-fixing legume, it directly enriches soil nitrogen, promoting plant growth. Concurrently, its dense, multi-stemmed canopy creates a favourable microclimate by reducing soil evaporation and temperature while protecting understory plants from wind. This dual action fosters higher understory biodiversity and cover, which in turn enhance soil organic matter input and stability, creating a self-reinforcing restoration cycle. The more limited impact of HS, despite its nitrogen-fixing capacity, suggests that this functional trait alone is insufficient for rapid ecosystem recovery. It is speculated that the sparser canopy and litter quality of HS may be less effective in modifying the microhabitat and building soil organic matter compared to CK. Consequently, the positive feedback loop between plant growth and soil improvement remained weaker. The root systems of CK and HS extend deeper in the vertical direction, whereas the root system of SP is mainly distributed within the 0–60 cm soil layer. Horizontally, it spreads widely, and its extensive roots effectively combine sand particles and improve soil porosity. However, the comparatively more open canopy of SP may offer less microhabitat amelioration than CK, resulting in a more modest promotion of understory vegetation and soil nutrients.

The random forest model provided strong, data-driven support for this mechanistic interpretation. The dominance of biodiversity indicators—particularly the Margalef index, perennial species richness and understory coverage—as primary predictors (*p* < 0.001) of GRA aligned with ecological theory positing that species richness and structural complexity enhance ecosystem multifunctionality (Maestre et al., 2012a, b). Specifically, the paramount role of the Margalef index underscores that taxonomic diversity, rather than dominance metrics (e.g., Simpson index), is critical for stabilising sandy ecosystems. This likely stems from niche complementarity: diverse perennial species assemblages in CK plantations maximise resource partitioning (e.g., water, light), thereby amplifying understory development and soil amelioration—consistent with the observed 209.67% cover increase under CK in the present study. The significant but secondary role of soil available phosphorus highlights its limiting nutrient status in arid soils. Unlike nitrogen (often mitigated by leguminous CK nitrogen fixation), phosphorus scarcity persists due to high fixation in calcareous sands (Mahdi and Mouhamad, 2018; Wahba et al., 2019). Based on these findings, it is recommended that future trials of CK × SP mixed plantations, which theoretically combine the superior microhabitat moderation and nitrogen fixation of CK with the superior soil-structuring capacity of SP, offer a promising strategy for achieving functional complementarity in sandy area restoration.

### 4.3 Limitations and future perspectives

This study was primarily conducted in the middle reaches of the Yellow River. Some other contemporary studies have also selected representative artificial forests in the middle and upper reaches of the Yellow River Basin for the comprehensive evaluation of their soil properties. Zhu et al. found that mixed coniferous and broad-leaved forests, larch forests, and mixed arbour and shrub forests also exhibited sound soil improvement effects (Zhu et al., 2023). Li et al. selected four typical mixed vegetation types in the sandy land of the Yellow River–desert transition zone, with the results showing that *Populus alba* var. *pyramidalis* × *C. korshinskii* was a suitable mixed vegetation restoration type in this region (Li et al., 2022). Although the dominance of shrubs is effective for rapid stabilisation, it may accelerate soil water consumption and suppress herbaceous biodiversity (Eldridge et al., 2011; Daryanto et al., 2019). Currently, the Chinese government advocates for the afforestation and management of planted forests based on the specific water resource conditions of different regions, particularly in areas with scant precipitation and severe water scarcity, thereby optimising the allocation of limited water resources. Investigating the relationship between water constraints and planted forest development will be a key research focus in the future. While the results of the present study demonstrate the effectiveness of shrub plantations (especially *C. korshinskii*) in enhancing vegetation development and soil quality, the need for adaptive management is emphasised. When establishing plantations in ecologically fragile areas, careful density control and mixed-species design are required to mitigate long-term hydrological and biodiversity costs.

This study provides a snapshot of the ecological effects of shrub plantations in the Kubuqi Desert. However, several limitations should be considered when interpreting the results. The findings are based on data from a single growing season. Given the dynamic nature of arid ecosystems, the observed patterns—particularly concerning soil moisture and nutrient dynamics—may not fully represent long-term trends or the stability of these restored ecosystems under inter-annual climate variability. Second, while the nested sampling design with 12 independent sites provides a robust baseline, the inherent heterogeneity of desert environments means that a larger sample size or a multi-year monitoring program could capture a broader range of ecological variation and strengthen the generalisability of this study’s conclusions. The random forest model effectively identified key drivers but primarily revealed correlative relationships. The underlying mechanisms—such as the specific plant-soil feedbacks, root exudate profiles or microclimatic modifications by different shrub canopies that lead to the superior performance of C. *korshinskii*—require further elucidation through controlled experiments. Integrating soil biological properties (e.g., microbial community structure) with the physicochemical measures taken here will be a critical next step to unravel these mechanisms. Additionally, this study did not involve arbour or mixed forests, and the selection of soil indicators was primarily based on physical and chemical properties. Research on biological characteristics still needs to be supplemented. Meanwhile, these results must be considered within the broader context of dryland restoration strategies. These deficiencies remain the direction for future research efforts.

## 5 Conclusions

Asteraceae, Fabaceae, Amaranthaceae and Poaceae accounted for 85% of the total plant species in the region. Asteraceae was the most adapted to the region and accounted for 30% of the total species, especially *Artemisia*, which accounted for 20% of the total species. Compared with bare sand, the soil clay of plantations increased by 1.67–6-fold, silt increased by 0.63–3.11-fold, water content increased by 23%–40%, total porosity increased by 36%–137%, total nutrient increased by 13%–369%, and available nutrient increased by 6%–181%, while soil bulk density decreased by 8%–13%. Soil fine particles and nutrients were mainly concentrated in the 0–20 cm soil layer, and the plantations had no significant effect on very coarse sand. In the 0–60 cm soil layer, soil nutrients and porosity decreased with increasing soil depth, while water content and bulk density showed the opposite change.

*Caragana korshinskii* plantations resulted in the highest vegetation diversity index. *C. korshinskii* plantations could better promote the accumulation of soil nutrients and fine particles, resulting in 1.4–2.67-fold more clay, 1.17–1.42-fold more silt, 1.19–1.75-fold more very fine sand, 1.05–2.9-fold more soil total nutrients and 1.01–2.4-fold more available nutrients than other plantations. *Salix psammophila* plantations were more effective at improving soil porosity structure and compactness in sandy areas, resulting in a 0.95–0.97-fold change in soil bulk density and a 1.06–1.18-fold increase in total porosity compared to other plantations. The evaluation results of the weighted GRA were: *C. korshinskii* (0.8574) > *S. psammophila* (0.7790) > *H. scoparium* (0.6883) > bare sand (0.5637). Species diversity and understory vegetation cover were the most critical factors affecting GRA. These findings underscore the importance of species selection for restoration outcomes. *C. korshinskii* is recommended as a priority species for enhancing ecosystem functions in this region. Additionally, future forest management practices should place greater emphasis on soil available phosphorus.

## Data Availability

The original contributions presented in the study are included in the article/supplementary material. Further inquiries can be directed to the corresponding author.
